# Near‐infrared light‐sensitive liposomes for enhanced plasmid DNA transfection

**DOI:** 10.1002/btm2.10020

**Published:** 2016-07-29

**Authors:** Christian Wiraja, Malathi Mathiyazhakan, Fatemeh Movahedi, Paul Kumar Upputuri, Yingying Cheng, Manojit Pramanik, Liang Yang, David Laurence Becker, Chenjie Xu

**Affiliations:** ^1^ School of Chemical and Biomedical Engineering Nanyang Technological University 70 Nanyang Drive Singapore 637457 Singapore; ^2^ Singapore Centre on Environmental Life Sciences Engineering (SCELSE) Nanyang Technological University 60 Nanyang Drive Singapore 637551 Singapore; ^3^ School of Biological Sciences, Division of Structural Biology and Biochemistry Nanyang Technological University Singapore 639798 Singapore; ^4^ Lee Kong Chian School of Medicine Nanyang Technological University 59 Nanyang Drive Singapore 636921 Singapore; ^5^ Institute of Medical Biology, Agency for Science Technology and Research (A*STAR), 8A‐Biomedical grove, Biopolis Singapore 138648 Singapore; ^6^ NTU‐Northwestern Institute for Nanomedicine, Nanyang Technological University 50 Nanyang Avenue Singapore 639798 Singapore

**Keywords:** gold nanostars, NIR‐responsive liposome, plasmid DNA delivery, skin fibroblast, topical delivery

## Abstract

Near‐infrared (NIR) light‐responsive liposomes are attractive carriers for targeted and controlled drug delivery to the superficial organ or tissue (e.g., skin). This work describes the development of NIR‐responsive liposomes by incorporating gold nanostars within liposomes composed of Phospholipon 90 g and cholesterol. Following cellular delivery, photothermal effect around the gold nanostar upon NIR stimulation induces microcavitation and liposome phase transition which consequently triggers the release of encapsulated molecules. Taking GFP plasmid as an example, we demonstrate enhanced gene transfection into fibroblasts following NIR treatment.

## 1 Introduction

Light‐responsive liposomes are attractive carriers for targeted and controlled drug delivery to the superficial tissues and organs (e.g., skin). To accommodate the optical window of biological tissues, liposomes sensitive to near‐infrared (NIR) lights (650–900 nm) are preferred.^1^, ^2^ A convenient way to synthesize such liposomes is to incorporate photosensitizing components within thermal‐responsive liposomes. These can be lipid‐like chemicals that undergo conformational change under laser irradiation or nanoparticles that absorb light and convert the photon energy into thermal energy.^3^, ^4^ Besides producing heat which may induce liposomes to undergo phase transition, triggered drug release is also facilitated by micro‐cavitation. As intense local temperature gradient occurring around the photosensitizer generates air bubbles, the rapid growth and subsequent collapse of bubbles disrupt the integrity of the liposomal membranes, allowing release of encapsulated drugs.^5^

Gold nanomaterials are one of the most attractive photosensitizing agents due to their biocompatibility and distinctive optical properties.^6^ The gold nanospheres or nanoparticles are usually more efficient in absorbing the light energy at UV‐visible range.^7^ Gold nanorod, nanoshell, and nanostars meanwhile have their maximal absorption at the visible‐NIR region due to their anisotropic distribution of surface electron layers.^8^, ^9^ For example, Au nanorods with maximal absorption at 780 nm were incorporated into liposomes to allow the visualization and controlled delivery of siRNA in vivo.^10^ Hollow gold nanoshells were physically entrapped or SH‐lipid linked onto liposomes for the cellular delivery of 6‐carboxyfluorescein under NIR‐triggered cavitation.^5^ As a relatively new construct however, there has not been any research to explore the utilization of gold nanostars (AuNSs) for the construction of NIR‐responsive liposomes, despite their extensive applications in biosensing and plasmon‐enhanced spectroscopy.^11^, ^12^, ^13^

**Figure 1:**
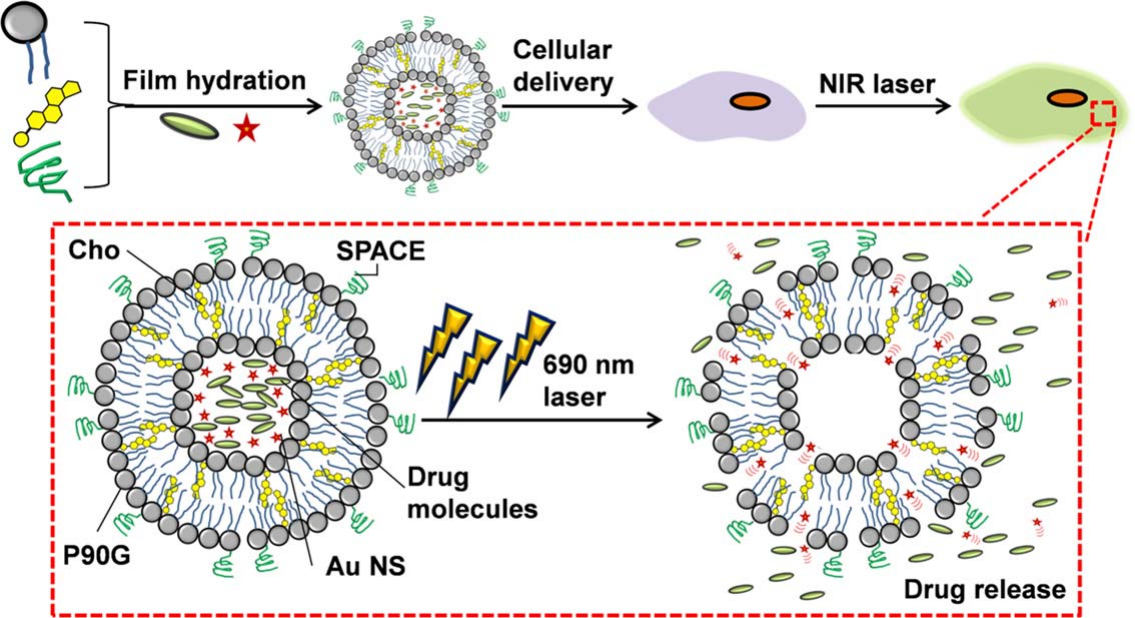
Schematic showing preparation and utilization of gold‐nanostars (AuNS)‐incorporated phospolipon 90G (P90G)/cholesterol (Cho) liposome for NIR‐triggered drug delivery. Upon laser stimulation at 690 nm, microcavitation occurs around AuNS which ruptures the liposome, facilitating effective unloading of encapsulated drugs

This article explores the development of a NIR‐responsive liposome by encapsulating AuNSs within liposomes composed of Phospholipon 90G (P90G) and cholesterol (Cho). P90G is a lecithin produced from soybean with great biocompatibility and natural presence in the human skin.^14^, ^15^ Cho is another skin lipid that is commonly incorporated in liposomes to improve its physical stability.^16^ To facilitate cellular internalization and the potential transdermal penetration, skin‐penetrating‐and‐cell‐entering (SPACE) peptides were conjugated onto the liposomes.^17^, ^18^ Calcein molecules or GFP‐encoding DNA plasmids were encapsulated within the liposomes together with the AuNSs during the film hydration step. Upon laser treatment, photothermal effect around the AuNSs induced the formation and collapse of vapor bubbles (micro‐cavitation), which disrupted liposome integrity and alongside the thermally‐induced phase transition triggered the release of encapsulated molecules (Figure 1).^5^, ^19^ To this end, the elevated cellular fluorescence following NIR treatment serves as an indicator of successful delivery and gene transfection.

## 2 Materials and methods

All materials except specifically mentioned were obtained from Sigma‐Aldrich (Singapore). P90G (composed of Phosphatidylcholine 94.4%, lysophophatidylcholine 2.5%, non‐polar lipids 1.5%, water 0.4%, tocopherol 0.2%, and ethanol 0.1%) was a gift from Lipoid (Lipoid, Switzerland). Cholesterol was purchased from Avanti (Avanti polar lipids Inc, USA). SPACE peptide (ACTGSTQHQCG, Disulfide Bridge 2–10) was purchased from RS Synthesis (USA).POPE‐NHS was obtained from NOF (USA). pEAK12‐GFP plasmid as described previously was amplified by transforming into DH5α‐competent cells and purified by gel electrophoresis.^20^, ^21^ PD10 column was obtained from GE Healthcare (Singapore). SnakeSkin^TM^ Dialysis Tubing and BSA protein assay kit were supplied by ThermoFisher Scientific (Singapore). Hoescht 33342 was purchased from Life Technologies (Singapore).

### 2.1 Synthesis of liposomes

Liposomes were synthesized according to the previously published protocol.^18^ Briefly, P90G and Cho were dissolved in 2 ml ethanol at different mole ratios (7:0, 7:1, 7:2, 7:7, 7:14, and 7:28) with fixed total weight (25 mg). For SPACE peptide conjugation, POPE‐NHS (5 mg/ml in ethanol) and SPACE peptide (5 mg/ml in phosphate‐buffered saline/PBS, pH 8) were added into the mixture following a 2 hr pre‐incubation at room temperature. Ethanol was evaporated using a rotary evaporator to form a thin film in a round‐bottom flask. The film was then hydrated with 1 ml calcein solution (200 µM) or DNA plasmid solution (150 ng/ml) pre‐mixed with 50 µl CaCl_2_ (50 mM) with magnetic stirring. pH of the solution was adjusted to 7.4 with 1 M NaOH solution. Un‐encapsulated dyes or plasmid were removed through PD10 column.

### 2.2 Synthesis of AuNSs and AuNS‐liposomes

AuNSs were synthesized by adding 50 µl gold precursor (HAuCl_4_, 20 mM) to 1 ml HEPES buffer (1 M, pH 7.4) under constant shaking (600 rpm) at room temperature.^19^ One hour later, AuNSs were purified through centrifugation (15k rpm for 30 min, 4°C) and rinsed with deionized water. The process was repeated thrice. AuNS‐liposomes were synthesized through hydrating the lipid film with a mixture of AuNSs and calcein or DNA plasmid solution. The subsequent steps were similar to the synthesis of liposomes.

### 2.3 Characterization of AuNS‐liposomes

#### 2.3.1 Encapsulation efficiency

The encapsulation efficiency of calcein or DNA plasmid was evaluated by disrupting the liposomes with 10% Triton X‐100 solution and quantifying the concentration of encapsulated molecules. Calcein was quantified through plotting the fluorescence signal of the disrupted liposome solution against a calcein fluorescence standard curve. DNA was quantified through measuring its absorbance at 260 nm (NanoDrop spectrophotometer) and plotting against the DNA absorbance standard curve.

#### 2.3.2 Release profile

Five hundred microliters of liposomal solution (5 mg/ml) was placed in dialysis tube (10 kDa molecular weight cut off, ThermoFisher Scientific) that was placed in 5 ml PBS (or PBS/fetal bovine serum‐FBS mixture) at 37°C and pH 7.4. At designated timepoints, 100 μl of the solution was sampled and replaced with fresh PBS. By measuring the fluorescence of the PBS solution and plotting against the fluorescence standard curve, the in situ concentration of calcein in the release buffer was derived. The release profile of DNA plasmid was quantified in a similar way except by measuring the absorbance of sampled solution at 260 nm.

#### 2.3.3 Liposome stability

The hydrodynamic size and polydispersity index (PDI) of liposomes were characterized with Zetasizer nano Z (Malvern). To examine their stability, both factors were tracked for one month at 4°C.

#### 2.3.4 Characterization of AuNSs

The morphology of synthesized AuNSs were examined with high‐resolution TEM (FEI G2 spirit, 120 kV). Ten microliters of aqueous solution containing AuNSs was placed on 200‐mesh formvar carbon‐coated copper grids (FCF‐200‐Cu) and dried in the dry cabinet for 72 hr before imaging.

#### 2.3.5 Photoacoustic (PA) spectrum acquisition

The PA excitation laser consists of an optical parametric oscillator (OPO) (Continuum, Surelite OPO) system pumped by a 532 nm Nd:YAG laser (Continuum, Surelite Ex). The OPO generates 5 ns duration pulses at 10 Hz repetition rate with tunable wavelength. AuNSs were irradiated with 680–920 nm lasers with 10 nm increments, and corresponding PA signals were collected using a 2.25 MHz UST (V323‐SU/2.25 MHz, Panametrics). Peak‐to‐peak PA signal was then normalized with the laser energy for each wavelength.

#### 2.3.6 Quantification of Au concentration

One hundred microliters of AuNS‐liposomes (5 mg/ml) were treated with 250 µl aqua‐regia overnight to dissolve the AuNSs. The solution was then diluted with 10 ml DI H_2_O. Inductively Coupled Plasma Spectrometer (ICP, Prodigy ICP spectrometer) analysis was used to determine the concentration of Au.

#### 2.3.7 Differential scanning calorimetry (DSC) analysis

Glass transition temperature for unloaded and AuNS‐loaded liposomes were analyzed using Pyris™ Diamond DSC (PerkinElmer). Briefly, ∼4–8 mg of liposome samples was loaded into aluminum pan for measurement. Systems were heated from 35 to 70°C at a rate of 5°C/min, and recorded heat flow was plotted after normalizing the sample weight.

### 2.4 Cell culture and liposome labeling

NIH‐3T3 (ATCC) were cultured in Dulbecco's Modified Eagle Medium (DMEM, high glucose 4.5 g/L and 4.0 mM l‐Glutamine) supplemented with 10% FBS and penicillin/streptomycin (100 U/ml) and kept at 37°C with 5% CO_2_. Cell labeling was performed by dispersing liposomes in DMEM with 1% FBS to a final liposome concentration of 5 mg/ml. Cells were incubated with liposome‐containing medium for 4 hr before being rinsed with PBS for further studies.

### 2.5 Fluorescence imaging

Fluorescence imaging was performed using a LX71 inverted fluorescence microscope (Olympus) and Retiga‐2000R CCD camera. The same settings (500 ms exposure time and 5× gain) were used to capture all green fluorescent images, at 100× image magnification. Finally, image processing with ImageJ was done to normalize background signal and quantify cellular intensity.

### 2.6 Flow cytometry

Flow cytometry analysis was performed by employing a LSR Fortessat X‐20 machine and accompanying FACSDiva software (BD Biosciences). For each sample, at least 10,000 cells were screened. Results were analyzed and plotted using FlowJo (TreeStar).

### 2.7 NIR laser stimulation

To evaluate laser‐induced calcein release from AuNS‐liposomes, liposome solution (5 mg/ml) were stimulated with pulsed OPO laser at 690 nm for 10, 20 or 40 s (10 pulse/s, with energy density of 10 mJ/cm^2^). Thirty minutes after, fluorescence of the supernatant was measured. In comparison, liposome solution was heated to 60°C for the same duration. For cellular laser exposure, 3T3 cells were seeded on a 48‐well plate and labeled with AuNS‐liposomes for 4 hr in culture medium containing 1% FBS. Stimulation with pulsed OPO laser at 690 nm was then performed for a total of 20 s per sample (10 pulse/s, with energy density of 10 or 40 mJ/cm^2^).

### 2.8 AlamarBlue viability assay

AlamarBlue viability assay was used to evaluate cytotoxicity of laser‐treated 3T3 (∼30k cells/well in a 48 well‐plate). AlamarBlue reagent was addeded into cell culture medium in 1–10 volume ratio for cell incubation. Twenty four hours later, fluorescence measurement (Ex: 570 nm/Em: 585 nm) was taken and normalized against untreated samples to measure the cell viability.

### 2.9 Statistical analysis

One‐way ANOVA was utilized to determine the significance in differences (*p* values), accompanied with Tukey post hoc analysis using the SPSS Statistics 20 software (IBM). Each experiment was performed by involving at least three replicates (*n* ≥ 3).

## 3 Results and discussion

### 3.1 Optimization of liposome composition to facilitate sustained release

Premature release of encapsulated drugs from the liposomal construct prior to cellular internalization can be detrimental towards its therapeutic efficacy.^22^, ^23^ Therefore, liposome (Lip) composition (i.e., ratio of P90G to Cho) was optimized to achieve the slowest release under normal physiological condition. By taking calcein as the model drug, we gradually increased the percentage of Cho from 0 to 80%. This increase significantly prolonged the time needed for the complete release of calcein from the liposomes (Lip‐calc; Supporting Information Figure S1A). The longest period of 96 hr was observed for liposomes composing of P90G and Cho with ratio of 7:28. This can be explained by the enhanced liposome rigidity, associated with less lipid bilayer exchange at greater Cho content.^16^ However, with the significant drop in encapsulation efficiency increasing Cho content further than 7:14 (Supporting Information Figure S1B), the composition was then fixed at 7:14 for the subsequent experiments. The presence of serum might influence the drug release profile.^24^ As shown in Supporting Information Figure S1C, the increase of FBS concentration in the labeling solution results in a faster release of calcein from liposomes. To minimize the cargo release during the labeling process and maintain the survival of cells, we chose medium containing 1% FBS as the labeling medium.

Finally, the release profile of GFP plasmid from liposomes was studied (Lip‐GFP; Supporting Information Figure S1D). In general, plamid‐liposomes and calcein‐liposomes have a similar release profile, suggesting that the release profile is dictated primarly by the composition of the liposomes and their dissolving environment.

### 3.2 Conjugation of SPACE peptides

To improve cellular uptake and the potential transdermal penetration, SPACE peptides were conjugated onto the liposomes (LipS). Since this modification was done on the surface of liposomes, it imposed minimal influence to the drug loading efficiency and long‐term stability of liposomes (Supporting Information Figure S2). The conjugation of SPACE peptide also did not compromise their stability at 4°C storage condition. However, the conjugation of SPACE peptides increased the size of liposomes from ∼130 to ∼170 nm (Supporting Information Figure S3A) and reduced the zeta potential from −25 to −70 mV (Supporting Information Figure S3B).

**Figure 2:**
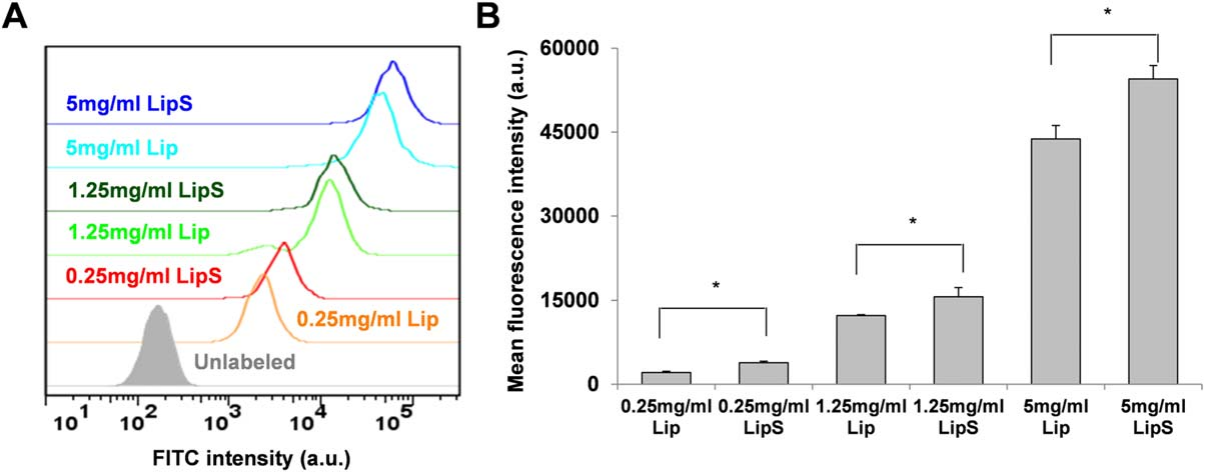
Calcein delivery with Lip and LipS. (A) Flow cytometry histogram plot showing cell fluorescence following liposomal labeling. (B) Mean fluorescence intensity following Lip & LipS labeling at designated concentrations. * represents *p* < .05

Then, we looked at the intracellular uptake of the liposomes following SPACE peptide conjugation through both fluorescence imaging and flow cytometry. The concentration factor was considered by labeling cells with liposomes at three different liposome concentrations (0.25, 1.25, and 5 mg/ml). Supporting Information Figure S4 shows representative cell images following calcein liposome labeling. For all the three concentrations, LipS‐labeled cells showed higher fluorescence intensity than Lip‐labeled cells (Figures 2A and 2B). At the lowest concentration of 0.25 mg/ml, the conjugation of SPACE peptide resulted in 36.5% increase in mean fluorescence intensity. Meanwhile, there were 27.6 and 24.5% increase at 1.25 and 5 mg/ml Lip concentration.

**Figure 3:**
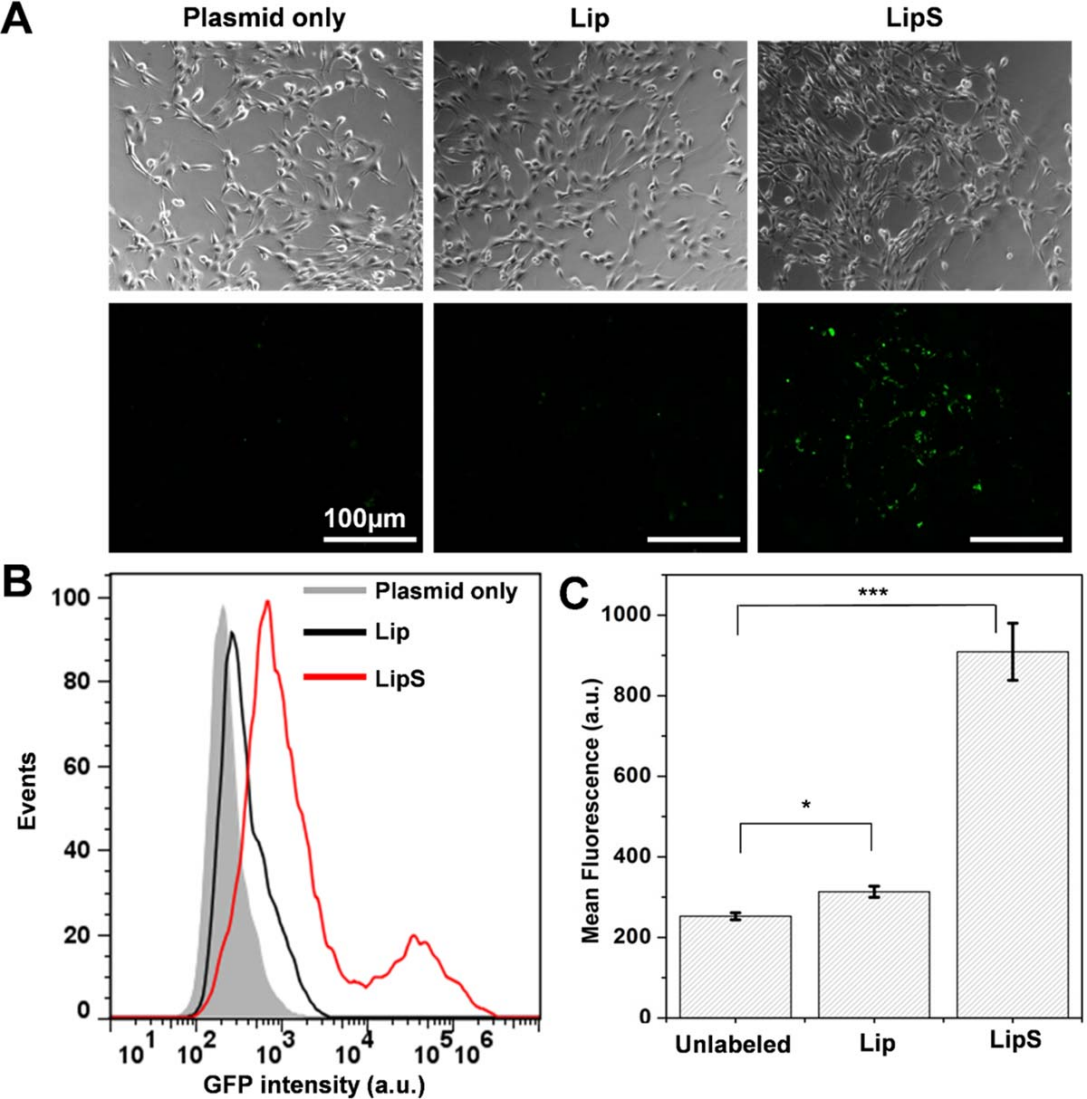
GFP plasmid delivery with Lip and SPACE‐conjugated Lip. (A) Representative images of cells following liposome labeling at 5 mg/ml. (B) Flow cytometry histogram and (C) mean fluorescence intensity of cells following plasmid transfection through Lip & LipS labeling. Scale bar = 100 µm. * represents *p* < .05, *** represents *p* < .001

Motivated by this observation, GFP plasmid was then encapsulated within LipS for cell transfection. As shown in Figure 3A, LipS‐GFP labeled cells became fluorescent while Lip‐GFP labeled cells were still comparable to unlabeled cells. Flow cytometry analysis further corroborated this finding with ∼3‐fold mean fluorescence intensity following 5 mg/ml LipS delivery, as compared to Lip‐treated cells (Figures 3B and 3C).

To this end, SPACE peptide conjugation indeed promoted cellular delivery of loaded molecules and plasmid.

### 3.3 Incorporation of AuNSs within liposomes for NIR‐triggered delivery

The AuNSs (45–50 nm in diameter, Supporting Information Figure S5A) were then incorporated into the liposomes during the thin film hydration step. ICP analysis revealed that 40.05 ± 0.532 µmol Au was incorporated per 5 mg of Lip, which roughly translates to 0.79 AuNS per Lip particle. The encapsulation efficiency of AuNS was ∼20%. This matches our expectation that most liposomes contain at least one AuNS. Photoacoustic spectroscopy shows that these AuNS‐containing liposomes (Lip Au) had the the highest radiation‐to‐acoustic conversion rate for light at 690 nm wavelength (Supporting Information Figure S5B). Thus laser at 690 nm was chosen for all the following experiments. Importantly, AuNS incorporation did not significantly alter the glass transition temperature of the liposome. DSC analysis revealed that the transition occurs at 48.75°C for unloaded liposomes, with only a 0.4°C difference for Lip Au (48.34°C; Supporting Information Figure S5C).

**Figure 4:**
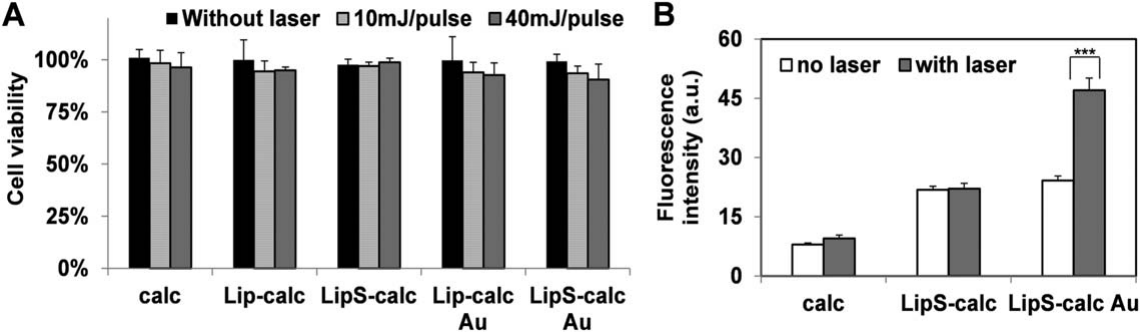
Laser‐triggered calcein delivery using AuNS‐containing Lip & LipS. (A) Alamar viability assay of 3T3 labeled with liposome carrying both calcein and AuNS after laser treatment. (B) Fluorescence signal quantification showing laser‐induced delivery of calcein in LipS Au. *** represents *p* < .001

Supporting Information Figure S6 is the procedure used to examine the controlled release of molecules from the AuNS‐liposomes with 690 nm laser. Laser settings were initially adjusted to minimize disturbance and toxicity to the cells, by taking into account Lip and LipS containing/not containing AuNS. As presented by Figure 4A, minimal cytotoxic effects were seen from all the groups tested, at both 10 mJ and 40 mJ pulse intensity (10 pulse/s, during a 20 s illumination). To be specific, ∼5% loss of cell viability was observed with 10 mJ pulse, while 40 mJ pulse resulted in ∼8–10% cell death. This loss of viability can at least be partially attributed to AuNS‐lip damaging endo/lyso‐somes enveloping them, with the occurance of local heating and micro‐cavitation during laser exposure. During this period, cytosolic acidification and release of endo/lysosomal enzymes may ultimately lead to cell death.^25^, ^26^ In view of this, laser exposure was set to 10 mJ/pulse in subsequent experiments.

The next step was to assess the NIR‐triggered release of calcein and GFP plasmid from liposomes. Testing on Lip solution (5 mg/ml) without involving cells, we noted steady increase of calcein released from the AuNS‐containing Lip as irradiation time was extended, reflected by the fluorescence intensity of the supernatant (Supporting Information Figure S7). In addition, we observed significantly lesser calcein release when the Lip solution was heated beyond its transition temperature (60°C) instead. This highlights the role microcavitation plays during the NIR‐triggered release, adding to the release induced thermally when liposomes underwent phase transition.

Thereafter, we evaluated the platform efficacy to achieve NIR‐triggered intracellular delivery. As quantified in Figure 4B and represented in Supporting Information Figure S8 for calcein loading, cell labeling with LipS and AuNS‐containing LipS (LipS Au) led to similar fluorescence intensity, unlike calcein only group which is not well internalized by the cells. Crucially after laser illumination, LipS Au‐labeled cells exhibited strong fluorescence which is not observed from both calcein only and LipS group. Specifically, 1.94‐fold stronger signal intensity was observed following laser stimulation. Meanwhile, laser activation does not significantly change the fluorescence in calcein only and LipS group (1.19 and 1.01‐fold, respectively). This demonstrates the effective laser‐triggered unloading of the proposed carrier. To substantiate this finding, we also compared calcein delivery from Lip and Lip Au (without SPACE peptide). As expected, only cells labeled with AuNS‐containing liposomes exhibited significantly stronger fluorescence after laser treatment (Supporting Information Figure S8).

**Figure 5:**
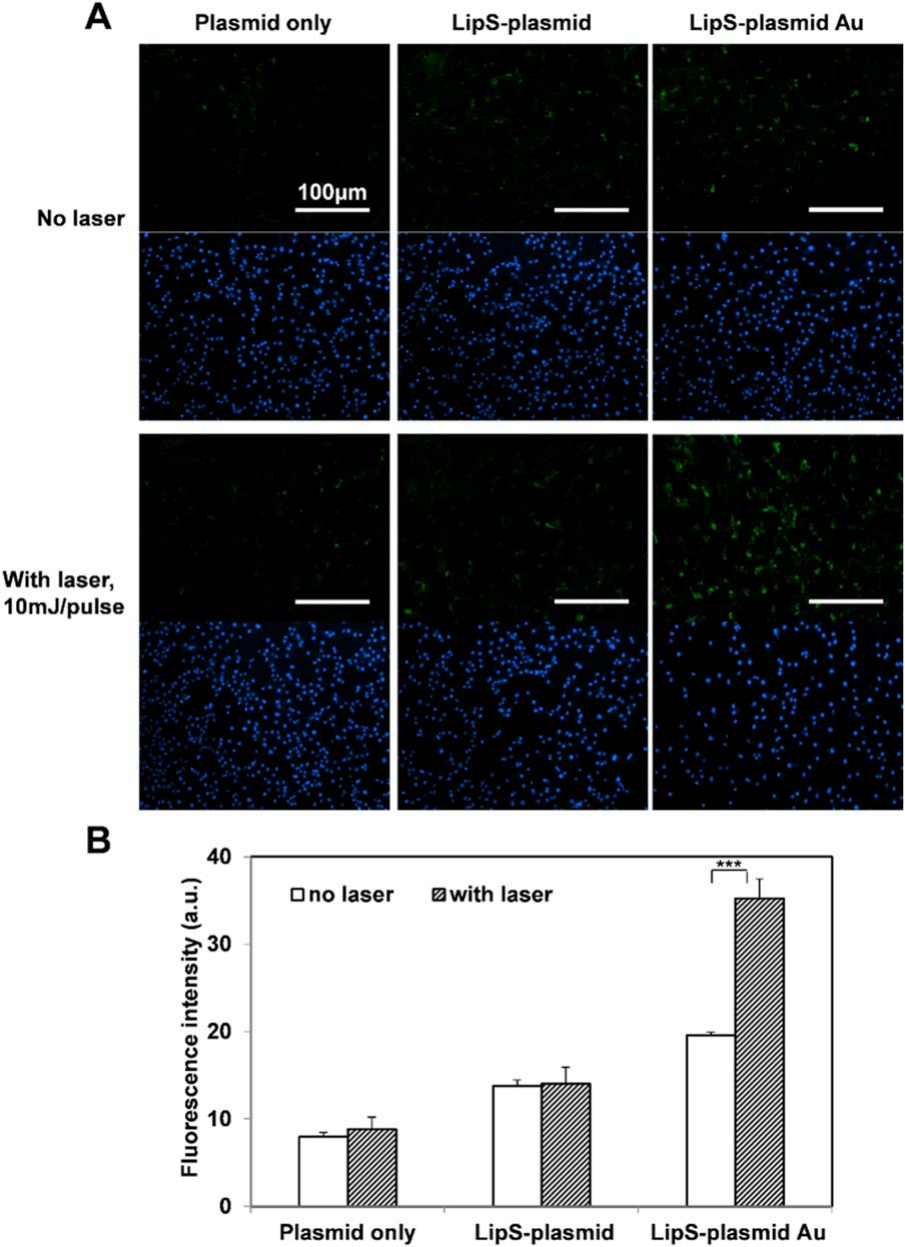
NIR laser‐stimulated GFP plasmid transfection using AuNS‐containing Lip/LipS. Representative GFP/nucblue fluorescence images (A) and signal quantification (B) revealed laser‐induced plasmid delivery. Scale bar = 100 µm. *** represents *p* < .001

Similar observation was found for GFP plasmid delivery (Figure 5). Without laser treatment, minimal GFP fluorescence can be observed from all the groups (plasmid only, LipS and LipS Au). Following laser activation however, significant signal enhancement was seen on LipS Au group (1.80‐fold). On the other hand, plasmid only and LipS group show insignificant GFP increase 24hrs post‐laser treatment (1.1 and 1.02‐fold, respectively).

Therefore, the proposed light‐responsive, SPACE‐modified liposome does enable controlled and versatile molecules delivery. In the future, even greater extent of release can be achieved by improving the AuNS incorporation with the liposomes. For example, AuNSs can also be tethered to the liposome surface employing thiol (SH)‐ended peptide/lipid linkers.^5^

## 4 Conclusion

Here, we report the construction of NIR‐responsive liposomes with AuNSs. Considering biodegradability and deformability, natural phospholipids (P90G) was used in conjunction with Cho as the basic components of the liposomes. To ensure minimal loss of molecules prior to cellular internalization while having optimal encapsulation, a composition of 7:14 mol ratio between P90G and Cho was chosen to provide slow, sustained release of both small (calcein) and big molecules (GFP plasmid). SPACE peptides were then conjugated to improve the cellular uptake of the liposomes. Furthermore, incorporation of AuNSs within the liposomes permits the conversion of NIR‐radiation energy to heat energy, leading to microcavitation and phase‐transition which disrupt the overall liposome integrity. Taking calcein and GFP plasmid as examples, we demonstrated the successful release of drugs within the cell cytoplasm after the NIR‐laser trigger. In the future, this system will be applied to animal models for the treatment of genetically‐related skin diseases.

## Supporting information

Additional supporting information may be found in the online version of this article at the publisher's web‐site.

Supporting Information
