# Supplementary material for: Near‐infrared light‐sensitive liposomes for enhanced plasmid DNA transfection
Source: Bioeng Transl Med. 2016 Jul 29;1(3):357–64. doi: 10.1002/btm2.10020 (PMC5689532; doi:10.1002/btm2.10020)
Supplement: Supplementary file 1 — Supporting Information [file BTM2-1-357-s001.pdf]

Supporting Information for

Near-infrared light-sensitive liposomes for enhanced plasmid DNA transfection

Christian Wiraja[1], Malathi Mathiyazhakan[1], Fatemeh Movahedi[1], Paul Kumar Upputuri[1], Yingying Cheng[2], Manojit Pramanik[1], Liang Yang[2,3], David Laurence Becker[4,5], Chenjie Xu[1,6*]

[1] School of Chemical and Biomedical Engineering, Nanyang Technological University, 70 Nanyang Drive, Singapore 637457, Singapore

[2] Singapore Centre on Environmental Life Sciences Engineering (SCELSE), Nanyang Technological University, 60 Nanyang Drive, Singapore 637551, Singapore

[3] School of Biological Sciences, Division of Structural Biology and Biochemistry, Nanyang Technological University, Singapore 639798, Singapore

[4] Lee Kong Chian School of Medicine, Nanyang Technological University, 59 Nanyang Drive, Singapore 636921, Singapore

[5] Institute of Medical Biology, Agency for Science Technology and Research (A*STAR), 8A-Biomedical grove, Biopolis, Singapore 138648, Singapore

[6] NTU-Northwestern Institute for Nanomedicine, Nanyang Technological University, 50 Nanyang Avenue, Singapore 639798, Singapore

* Correspondence should be addressed to X.C.J. (cjxu@ntu.edu.sg)

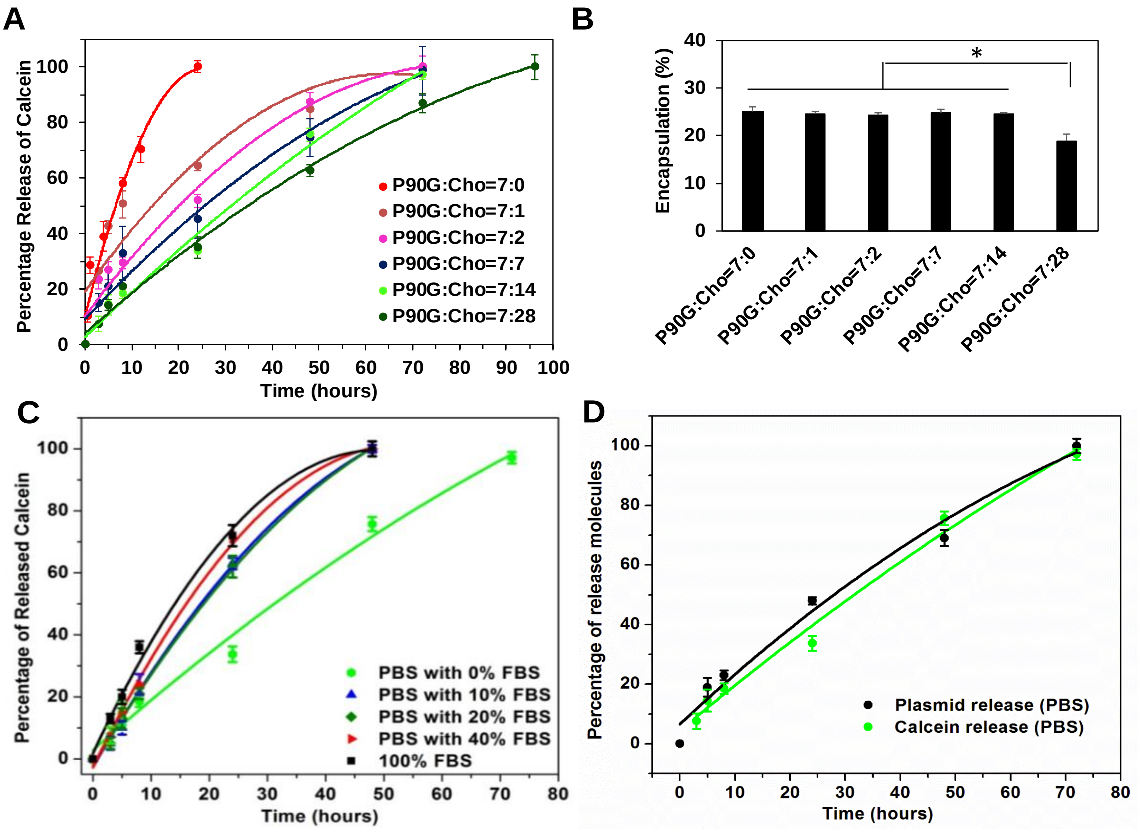

Figure.S1: Optimization of the liposome composition; A) influence of composition to calcein release profile. B) Encapsulation efficiency of calcein on liposomes with different compositions. C) The influence of serum concentration towards calcein release profile from optimized liposome. D) Release profile of plasmid and calcein from optimized liposome in PBS. * represents P<0.05.

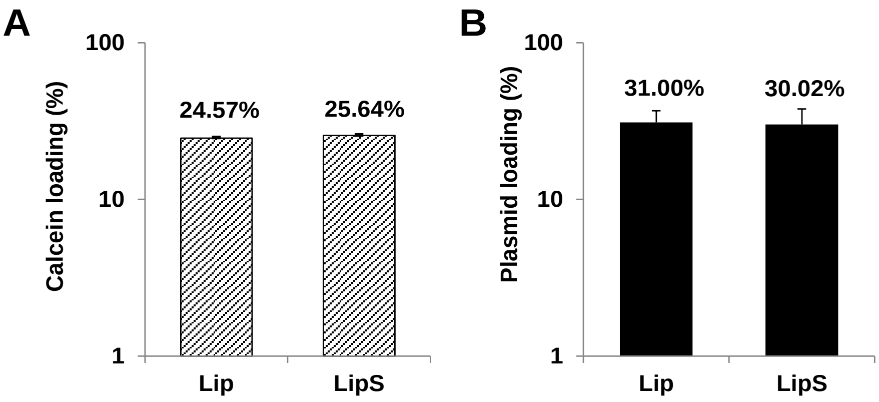

Figure.S2: Lip and Lip+SPACE (LipS) loading efficiency comparison for calcein (A) & GFP plasmid (B).

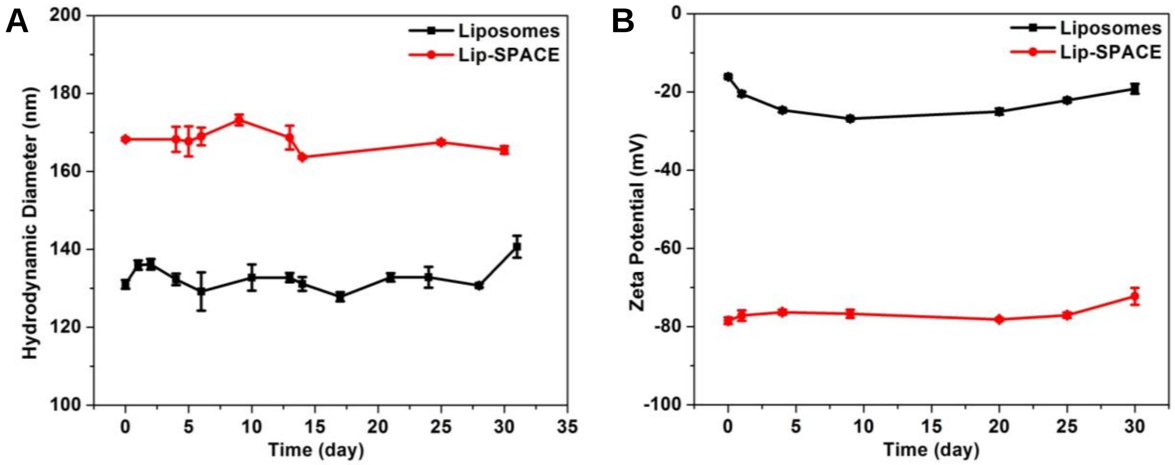

Figure.S3: Lip and LipS stability characterization. A) Hydrodynamic diameter and B) zeta potential changes of liposomes over a month.

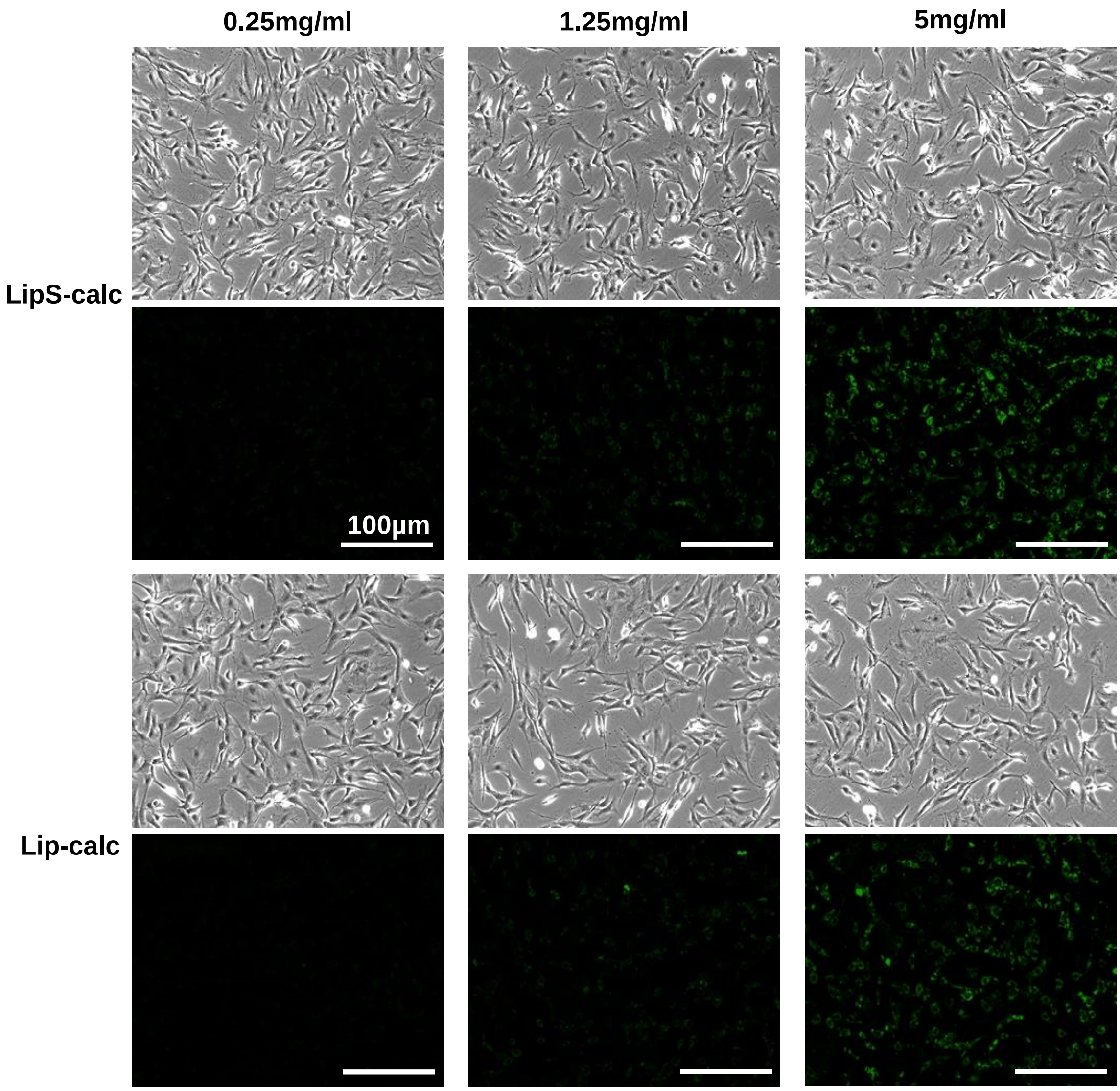

Figure.S4: Representative fluorescence images showing calcein delivery by Lip and LipS at various concentration (0.25, 1.25 & 5 mg/ml). Scale bar=100 µm.

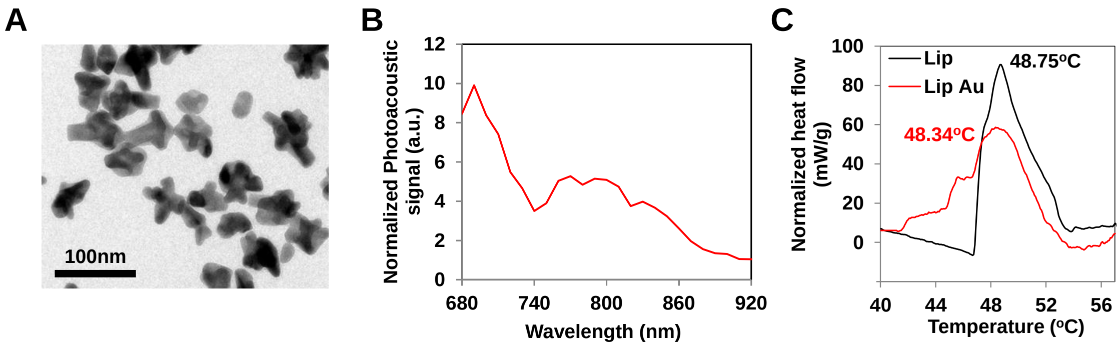

Figure.S5: Characterization of AuNS and AuNS-loaded liposomes. A) TEM images of synthesized AuNS for liposomes incorporation. B) Photoacoustic spectra of AuNS showing signal peak at 690 nm. C) DSC analysis showing glass transition temperature of Lip and AuNS-incorporated Lip. Scale bar=100 nm.

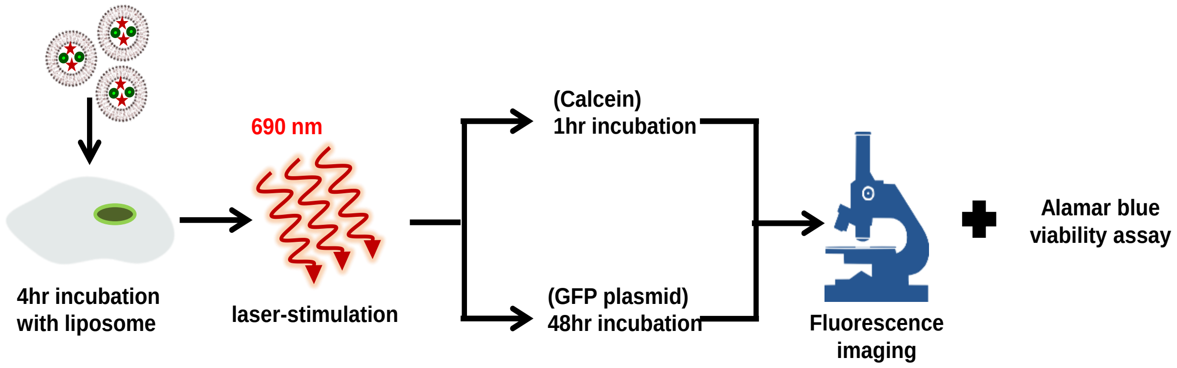

Figure.S6: Schematic showing procedures involved in near infrared-stimulated Lip/LipS-AuNS delivery.

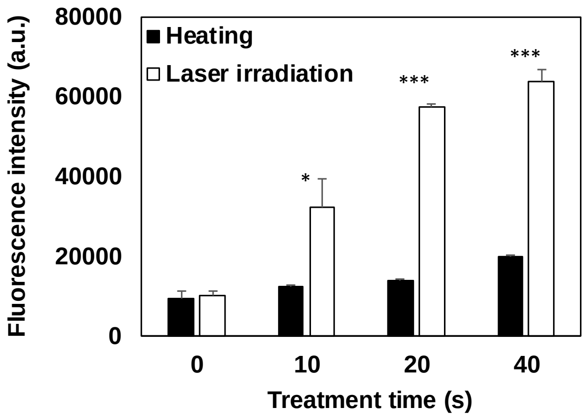

Figure.S7: Calcein release from Lip-calc Au post 60 ºC heating or laser irradiation (10 mJ/pulse) at different treatment periods. * represents P<0.05, * represents P<0.001.

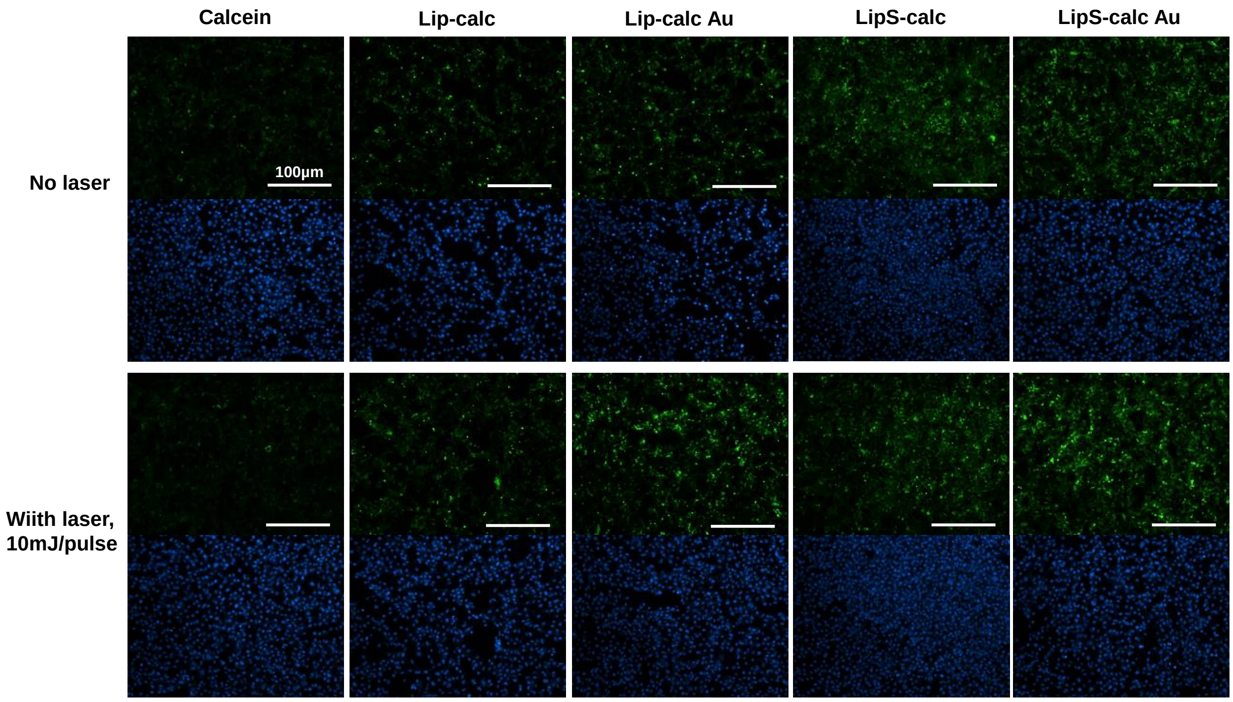

Figure.S8: Representative images showing NIR laser-stimulated calcein delivery with AuNS-containing Lip and LipS. Scale bar = 100 µm.
